# Lignin and cellulose dynamics with straw incorporation in two contrasting cropping soils

**DOI:** 10.1038/s41598-018-20134-5

**Published:** 2018-01-26

**Authors:** Xiangbi Chen, Yajun Hu, Shuzhen Feng, Yichao Rui, Zhenhua Zhang, Hongbo He, Xinhua He, Tida Ge, Jinshui Wu, Yirong Su

**Affiliations:** 1 Key Laboratory of Agro-ecological Processes in Subtropical Region, Institute of Subtropical Agriculture, the Chinese Academy of Sciences, Changsha, 410125 P.R. China; 2 Southern Regional Collaborative Innovation Center for Grain and Oil Crops in China, Hunan Agricultural University, Changsha, 410128 P.R. China; 3 Guangxi University of Science and Technology, Liuzhou, 545006 P.R. China; 4 Department of Soil Science, University of Wisconsin-Madison, Madison, WI 53706 USA; 5 Key Laboratory of Terrestrial Ecological Process, Institute of Applied Ecology, the Chinese Academy of Sciences, Shenyang, 110016 P.R. China; 6 Centre of Excellence for Soil Biology, College of Resources and Environment, Southwest University, Chongqing, 400715 P.R. China

## Abstract

Incorporation of crop residues is essential to enhance soil organic matter in arable ecosystems. Here, we monitored the dynamics of cellulose and lignin, the most abundant constituents of plant residues, and their relationships with enzyme activities, microbial gene abundances and soil properties after 13-year long-term and one-year short-term crop straw incorporation into upland and upland-paddy soils in a field-based experiment. Lignin, rather than cellulose, accumulated in both soils following straw incorporation. Cellulose was almost completely converted into non-cellulose forms within 6 and 3 months after straw incorporation into upland and upland-paddy rotation soils, respectively. Whereas, lignin accumulated at the rate of 129 and 137 mg kg^−1^ yr^−1^ within 13 years’ straw incorporation in upland and upland-paddy rotation, respectively. The predominance of recalcitrant vanillyl monomers in upland-paddy rotation indicated a high stability of lignin. Structural equation models revealed that the key factor driving cellulose and lignin dynamics was available nitrogen, followed by enzymes activities (cellobiohydrolases and laccases) and functional genes abundances (*cbh*I and *laccase*-like) as mediated by soil pH. Our findings highlighted that upland might have higher carbon sequestration rate, whereas upland-paddy rotation system was more beneficial for accumulation of recalcitrant organic fractions under crop residue incorporation.

## Introduction

China is one of the major rice producers in the world, with 26% of its total cultivated land growing rice (~30 Mha) (National Bureau of Statistics of China, 2008). About 86% of China’s rice production is cultivated under upland-paddy rotation system (e.g. wheat-rice, maize-rice, rapeseed-rice)^1,2^, which is also important in other Southeast Asian countries such as Bangladesh, India, Nepal, and Pakistan^3^. The upland-paddy rotation system is different from upland, where crops are grown in well-drained soil under aerobic conditions, or paddy soils, where soil is puddled before rice transplanting and kept flooded to create anaerobic conditions during rice growth. In the upland-paddy rotation system, the number and activity of oxidizing bacteria are decreasing during the paddy season, which lowers decomposition of soil organic matter (SOM) and contributes to SOM accumulation^3^; however, during upland crop season, a significant SOM loss was observed due to quick SOM decomposition^4^. Although the alteration of drying and wetting process generally accelerates SOM decomposition^5^, evidence is still limited and controversial results have been reported regarding the accumulation rates of SOM in the upland and upland-paddy systems. For example, the carbon (C) accumulation rate is about three times higher in rice–wheat than in maize–wheat in India^6^, whereas in subtropical region of China where the red soil (Ultisol) is the main soil type, the upland-paddy rotation system (0.24 t ha^−1^ yr^−1^) has lower C accumulation rate than in upland (0.68 t ha^−1^ yr^−1^)^7^.

External organic matter input, such as the incorporation of crop residues, has been regarded as an effective practice to maintain SOM level in agricultural systems^8–10^, including in the red soils^11,12^. As the first and second most abundant constituents of vascular-plant tissues, cellulose and lignin together constitute 20–60% of the crop residues^13,14^, thus their decomposition rates largely determine the pool size of SOM^15^. Cellulose is generally considered to be more labile and is usually decomposed faster than lignin^16^ due to its chemical composition and structure^17^. However, it has been argued that the stability and low degradability of lignin in soils might have been overestimated which means its contribution to SOM pool can be exaggerated^15^. Indeed, various observations in the field suggested the decrease of lignin was similar or higher than cellulose^16^ or that of the bulk SOM^18,19^. Some studies based on NMR characterization of SOM also failed to detect an enrichment of lignin-derived aromatic structures in the mineral soil compared to the litter layers of forest soils^20–22^, or in stabilized fractions of SOM present in subsoil horizons^23^, reflecting no preferential stabilization of lignin in soils. Evidence, however, is limited to show that the inconsistent results of lignin stability among various ecosystems are related to the soil properties, especially soil C level and microbial biomass. The soils containing higher organic C which increased the microbial assimilation of lignin-derived C were more beneficial for the accumulation of lignin-derived C^16,18^.

The transformations of either cellulose or lignin are complex processes, which are carried out by microbes in the soil. A series of extracellular enzymes, such as endoglucanases, cellobiohydrolases and β-glucosidases are involved in cellulose degradation^24,25^, while laccases, lignin peroxidases and manganese peroxidases are involved in lignin degradation^26^. The *cbh*I gene that encodes cellobiohydrolase is considered to participate in the rate-limiting step in the decomposition of cellulose^24^. Laccases genes are proved to be widely distributed among virtually all bacterial phyla, and the abundance of the bacterial *laccase*-like gene is proportional to laccases activity in subtropical arable soils^27^. Thus, cellulose and lignin dynamics in soils can be reflected by the activity of cellobiohydrolase and laccase and abundances of their encoding functional genes, i.e. *cbh*I and *laccase*-like. However, very limited information is available on the abundance or activity of cellulose or lignin degradation microorganisms in upland or upland-paddy systems.

The red soils in subtropical China are characterized by low SOM and nutrients (N, P, S, etc.) levels, poor physical structure, and high acidity, due to the hot and humid climate^28^. Maintaining and improving SOM level is crucial to the physical, chemical and biological functions of cropping soils^29,30^. Here in a field experiment with 13-year long-term (from 2000 to 2013) and one-year short-term (from April 2013 to April 2014) incorporation of straw into soils in two contrasting upland and upland-paddy rotation systems in subtropical China, we studied the dynamics of lignin and cellulose, the activities of extracellular enzymes and the abundances of functional genes that were associated with lignin and cellulose transformations. The underlying hypotheses are as follows: 1) straw incorporation favors the accumulation of lignin but not cellulose due to their chemical nature; 2) cellulose and lignin will be accumulated in higher portions in the uplands compared to the upland-paddy soils due to higher C accumulation rate in the first one ecosystems^7^; 3) if hypothesis 2 is the case, the activities of functional enzymes and the abundances of microbial genes which were associated with lignin and cellulose transformation will be higher in upland-paddy rotation than in upland soils.

## Results

**Figure 1:**
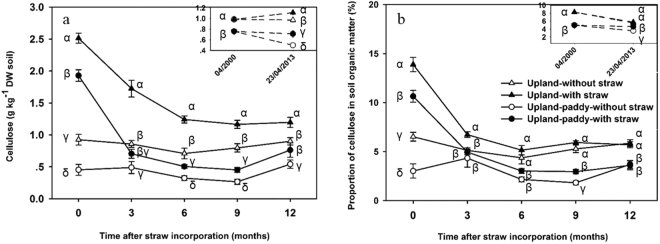
Dynamics of cellulose content (**a**) and its proportion in soil organic matter (**b**) (means ± SE, n = 4). 04/2000, the time before the field experiment started; 23/04/2013, the day before straw incorporation in 2013. Different *Greek letters* indicate significant difference among treatments at each sampling time at *P* < *0.05* level (according to one-way-ANOVA and LSD test).

Before establishing the field experiment in 2000, the initial cellulose in soil was significantly higher in upland (accounting for 8.2% of SOM) than that in upland-paddy (accounting for 5.0% of SOM) (Fig. 1). Thirteen years after the establishment of the experiment, the cellulose content was unchangeable in the upland soil, irrespective of the straw incorporation status, but a significant decrease under the without straw treatment in the upland-paddy soil was detected (Fig. 1a). Meanwhile, the proportions of cellulose in SOM were significantly decreased in upland and remained unchanged in upland-paddy after 13 years (Fig. 1b).

Over a one-year period following straw incorporation, both the cellulose content in soil and its proportion in SOM showed a similar dynamic in both systems (Fig. 1a and b). Meanwhile, the cellulose content in soil and its proportion in SOM were significantly greater in treatment with straw than without straw in both systems. Specifically, after a three-day of a half annual rate of straw incorporation in April 2013, the cellulose content rapidly reached 2.5 and 1.9 g kg^−1^, accounting for 14.0% and 12.0% of total SOM in the upland and upland-paddy soils, respectively. After three months, 56% and 100% of the added cellulose from straw was converted into non-cellulose forms in the upland and upland-paddy, respectively. Furthermore, after six months, 90% of the newly added cellulose was converted into other forms in the upland. At the end of the one-year period, the cellulose level for both with and without straw treatments maintained its initial level (Fig. 1).

**Figure 2:**
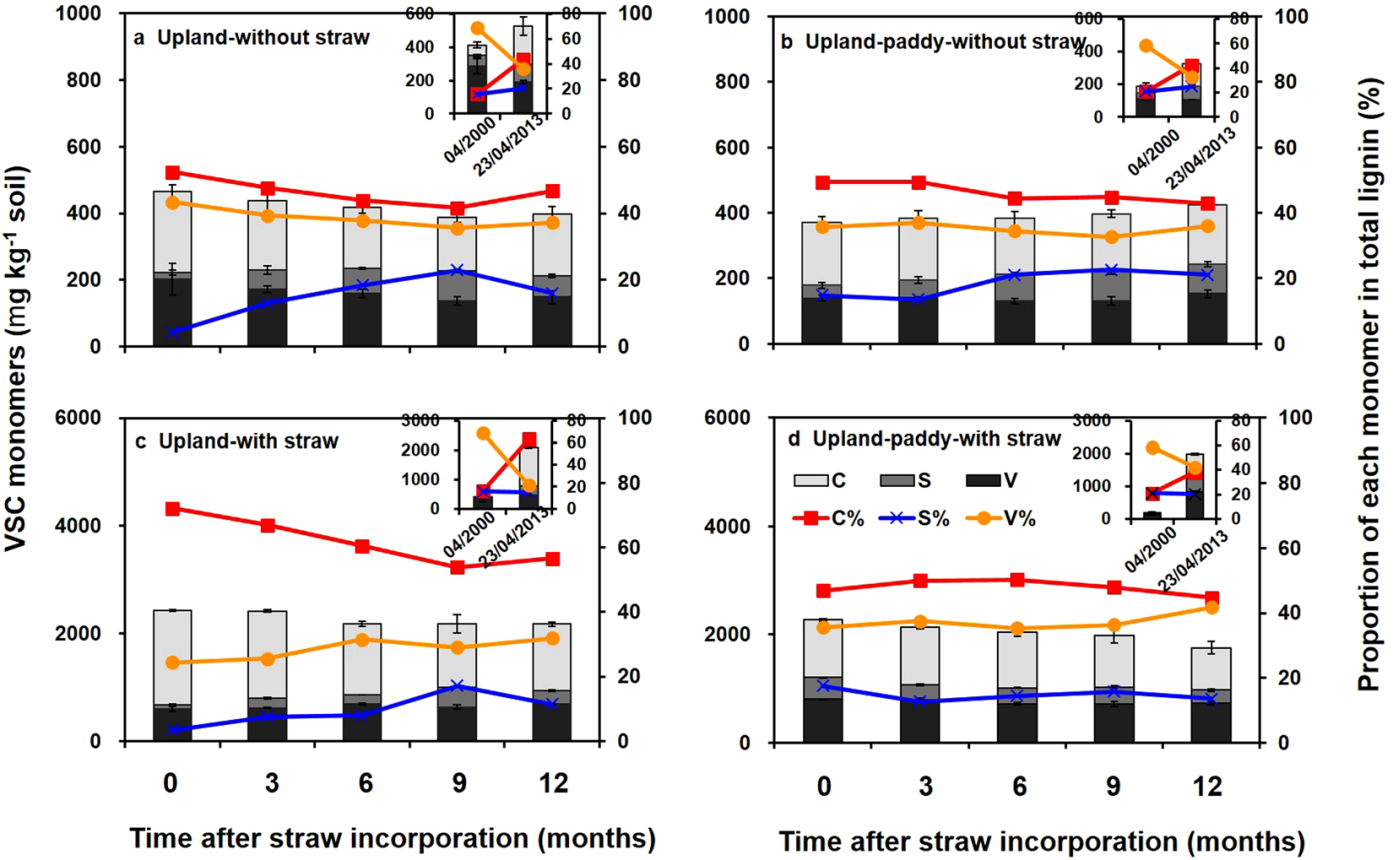
Dynamics of VSC monomers (i.e. lignin content) and the proportion of each monomer in total lignin (means ± SE, n = 4). Total lignin content was indicated as the sum of vanillyl (V), syringyl (S), and cinnamyl (C) type monomers (VSC) released from lignin macromolecules. 04/2000, the time before the field experiment was set; 23/04/2013, the day before straw incorporation in 2013.

**Figure 3:**
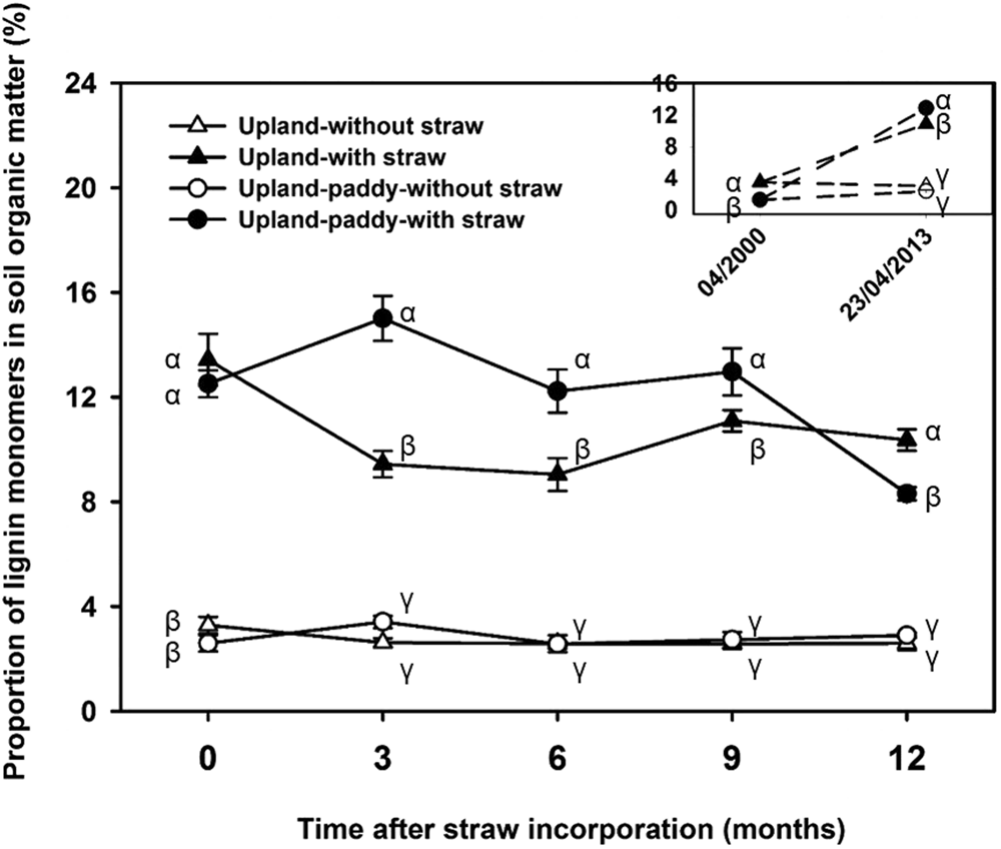
Dynamics of lignin proportion in soil organic matter (means ± SE, n = 4). 04/2000, the time before the field experiment started; 23/04/2013, the day before straw incorporation in 2013. Different *Greek letters* indicate significant difference among treatments at each sampling time at *P* < *0.05* level (according to one-way-ANOVA and LSD test).

In 2000, the lignin content, i.e. the VSC monomers, in the upland (415 mg kg^−1^, accounting for 3.5% of SOM) was significantly higher than that in the upland-paddy (189 mg kg^−1^, 1.2% of SOM; Figs 2 and 3). In 2013, lignin contents in the upland and upland-paddy increased to 529 and 325 mg kg^−1^ for treatments without straw incorporation, and to 2092 mg kg^−1^ (10.9% of SOM) and 1972 mg kg^−1^ (12.8% of SOM) for treatments with straw incorporation, respectively (Figs 2 and 3). Thus, the annual accumulation rates of lignin were 129 and 137 mg kg^−1^ in upland and upland-paddy, respectively, with its proportion in SOM increased by 3.1- to 10.4-fold with 13-year’ straw incorporation (Figs 2 and 3). After straw incorporation, the lignin contents temporarily reached 2432 and 2269 mg kg^−1^ in upland and upland-paddy, respectively (Fig. 2). Meanwhile, over a one-year period, both the lignin contents and proportion of each monomer in the total lignin displayed a similar unchanged status.

In 2000, the lignin in both upland and upland-paddy was dominated by the V-type monomer (Fig. 2). After the long-term and short-term straw incorporation, the lignin was dominated by the C-type monomer in upland while by both V- and C-type monomers in upland-paddy with V-type monomers decreased and C-type monomer increased (Fig. 2).

**Figure 4:**
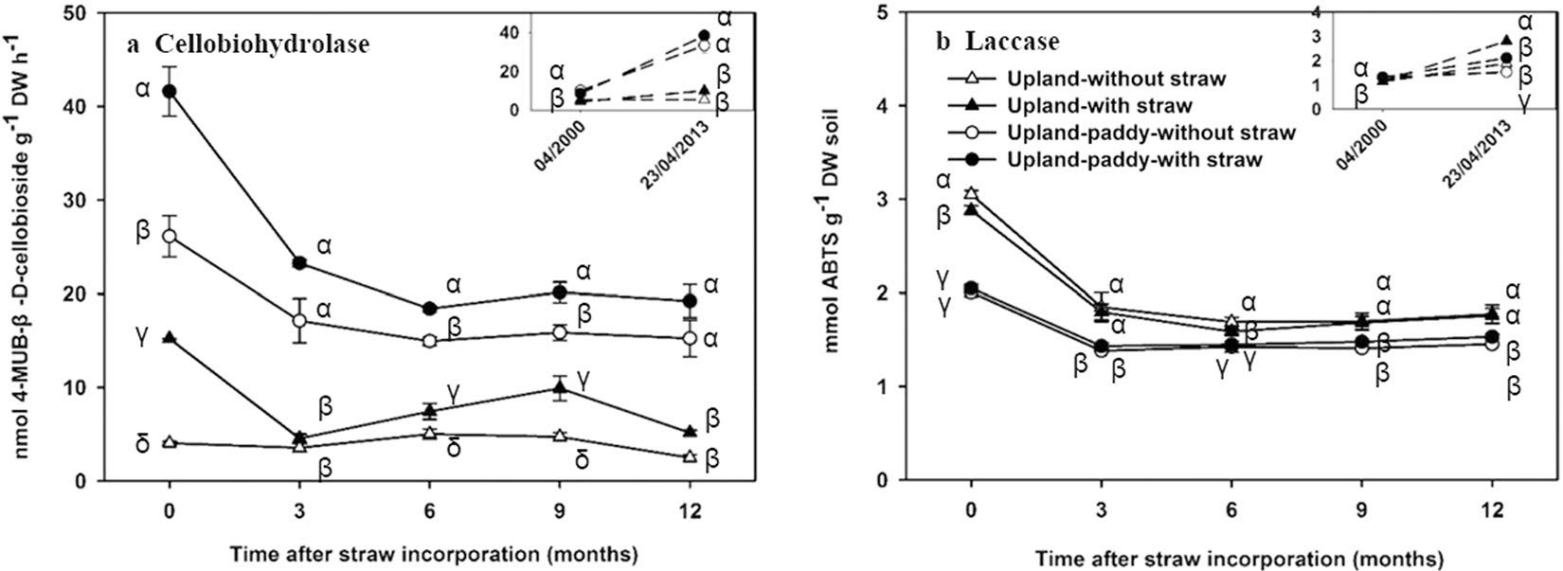
Dynamics of the activities of cellobiohydrolase (**a**) and laccase (**b**) (means ± SE, n = 4). 04/2000, the time before the field experiment was set; 23/04/2013, the day before straw incorporation in 2013. Different *Greek letters* indicate significant difference among treatments at each sampling time at *P* < *0.05* level (according to one-way-ANOVA and LSD test).

The cellobiohydrolases activity was significantly higher in upland-paddy than in upland and was higher for treatments with straw than without straw (Fig. 4). The laccases activity was generally higher in upland than in upland-paddy and was not significantly different between the treatments without and with straw (Fig. 4). The abundances of *cbh*I gene and *laccase*-like gene were irregularly fluctuated among treatments (Fig. S1).

**Figure 5:**
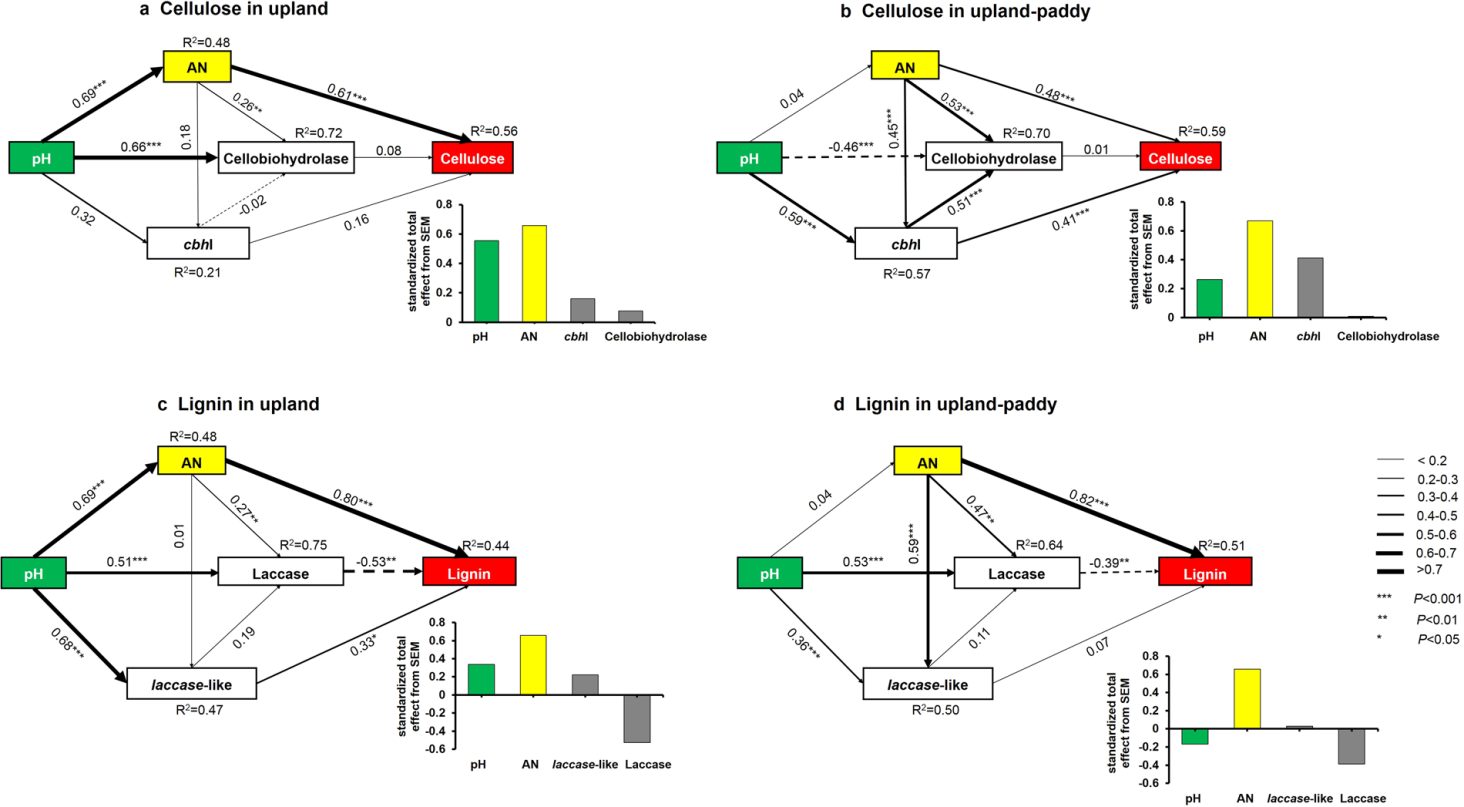
The structural equation model (SEM) showing the relationships among dynamic of cellulose or lignin, enzyme activity, microbial gene abundance, pH, available nitrogen (AN) in upland and upland-paddy rotation land, and their standardized total effects (direct plus indirect effects) derived from the SEMs (shown in the right corner of sub-figures). The width of the arrows indicates the strength of the standardized path coefficient. The solid lines indicate positive path coefficients and dashed lines indicate negative path coefficients. R^2^ values represent the proportion of the variance explained for each endogenous variable.

The structural equation model (SEM) was used to assess the extent of direct and indirect effects of explanatory variables on contents of cellulose and lignin in upland and upland-paddy rotation systems (Fig. 5). All of the four SEMs exhibited a reasonable fit based on our hypothesis (Table S1). The SEMs could explain 44–59% of the variance in cellulose or lignin dynamics, which depended on available nitrogen (AN), pH, enzyme activity and microbial functional gene abundance (Fig. 5).

For cellulose in upland, its dynamic was directly affected by AN (path coefficient is 0.61), and indirectly by soil pH (Fig. 5a). In upland-paddy, AN and *cbh*I gene abundance were identified as significant direct drivers of cellulose dynamic, with the path coefficients of 0.48 and 0.41, respectively, and pH was the indirect factor via influencing the *cbh*I gene abundance (Fig. 5b).

The dynamics of lignin in the upland soil were also directly mediated by AN, *laccase*-like gene abundance and laccase activity with the path coefficients of 0.80, 0.33 and −0.53, respectively, and indirectly by pH (Fig. 5c). In upland-paddy, AN and laccase activity were identified as significant direct drivers of lignin dynamic, with the path coefficients of 0.82 and −0.39, respectively, and pH was the indirect factor via influencing the laccase activity (Fig. 5d).

Standardized total effects derived from the SEMs revealed that cellulose was mainly driven by AN, followed by pH in upland, or followed by *cbh*I gene abundance in upland-paddy. For lignin in both systems, it was mainly driven by AN, followed by the negative effect on laccase activity (Fig. 5).

## Discussion

Thirteen years after the straw incorporation, the SOM content significantly increased in upland but maintained its initial level in upland-paddy rotation system (Table 1). Simultaneously, cellulose content and its proportion in SOM remained constant or decreased in upland and upland-paddy rotation (Fig. 1), indicating that cellulose did not accumulate in both systems. Studies have shown that the decomposition products of cellulose during initial stages, e.g. dissolved organic matter (accounting for 11–19%^31^), can be incorporated into microbial biomass at high rate resulting in efficient SOM formation^32^. Although the fates of cellulose could not be traced in our study, the significantly increased microbial biomass after straw incorporation (Fig. S2) was positively related to the content of cellulose, reflecting the close relationship between microbial biomass formation and cellulose content in the soil. Thus, the cellulose from newly-added straw did not accumulate as the original cellulose form but may contribute the SOM stock as other non-cellulose forms because the formation of organo-mineral association (microbial products with minerals) has been recognized as the main form of SOM stabilization^32,33^.

**Table 1:** Basic soil physicochemical properties before and after long-term straw incorporation^*^.

Treatments	pH		SOM (g kg^−1^)		TN (g kg^−1^)		TP (g kg^−1^)		TK (g kg^−1^)		AN (mg kg^−1^)		AP (mg kg^−1^)		AK (mg kg^−1^)		C/N	
	2000	2013	2000	2013	2000	2013	2000	2013	2000	2013	2000	2013	2000	2013	2000	2013	2000	2013
Upland-without straw	5.0 ± 0.0β	5.2 ± 0.0γ	11.9 ± 0.3β	17.5 ± 0.4β	0.7 ± 0.0β	0.9 ± 0.0β	0.5 ± 0.0β	0.5 ± 0.0β	20.6 ± 0.3α	20.7 ± 0.5α	148.9 ± 0.7β	402.0 ± 33.9β	5.0 ± 0.2β	8.4 ± 1.2β	69.8 ± 2.3α	207.3 ± 51.1α	10.3 ± 0.2β	11.1 ± 0.1α
Upland-with straw	5.0 ± 0.0β	5.3 ± 0.1βγ	11.9 ± 0.3β	19.3 ± 0.3α	0.7 ± 0.0β	1.0 ± 0.0α	0.5 ± 0.0β	0.5 ± 0.0β	20.6 ± 0.3α	19.9 ± 0.7αβ	148.9 ± 0.7β	628.5 ± 19.3α	5.0 ± 0.2β	8.0 ± 0.9β	69.8 ± 2.3α	139.2 ± 7.7βγ	10.3 ± 0.2β	11.0 ± 0.2α
Upland-paddy-without straw	5.7 ± 0.1α	5.9 ± 0.2α	15.2 ± 0.0α	13.9 ± 0.1δ	0.8 ± 0.0α	0.9 ± 0.0β	0.7 ± 0.0α	0.6 ± 0.0α	18.2 ± 0.1β	18.5 ± 0.1β	153.9 ± 0.9α	417.9 ± 33.7β	8.6 ± 0.5α	13.3 ± 0.4α	39.8 ± 1.2β	109.6 ± 9.8γ	11.6 ± 0.2α	8.9 ± 0.1β
Upland-paddy-with straw	5.7 ± 0.1α	5.5 ± 0.2β	15.2 ± 0.0α	15.4 ± 0.3γ	0.8 ± 0.0α	1.0 ± 0.0α	0.7 ± 0.0α	0.7 ± 0.0α	18.2 ± 0.1β	19.6 ± 0.3αβ	153.9 ± 0.9α	583.6 ± 12.8α	8.6 ± 0.5α	13.1 ± 0.6α	39.8 ± 1.2β	156.6 ± 13.4β	11.6 ± 0.2α	8.9 ± 0.5β

^*^2000 and 2013 represent the soil properties in 04/2000 (Month/Year) and before straw addition to soil on 23/04/2013 (Date/Month/Year), respectively (means ± SE, n = 4). Data followed by the same *Greek letter* in the same column are not significantly different (*P* < 0.05) among treatments. SOM, soil organic matter; TN, total nitrogen; TP, total phosphorus; TK, total potassium; AN, available nitrogen; AP, available phosphorus; AK, available potassium.

Although the cellulose content temporarily increased to 12−14% of the SOM after fresh straw was added, it may convert into other forms, e.g. CO_2_, microbial biomass C, dissolved organic C, within 3 months in the upland-paddy and within 6 months in upland soil (Fig. 1), suggesting a relatively quick transformation of cellulose in these soils. Upland-paddy rotation system is associated with frequent repetition of wetting and drying switched between anaerobic and aerobic conditions^3^ that will result in the significantly higher activity of cellulose-degrading enzymes (Fig. 4a), thus the rapid response of cellulose-degrading microorganisms after straw incorporation into upland-paddy (Figs S1 and S2) contributes to the faster loss of cellulose. The loss rates of cellulose in our tested systems were higher than those in highly degraded Calcaric regosol soils (9.3% was mineralized within 30 days)^16^, and anaerobic sediments (half of cellulose was degraded within 18 months)^34^. The higher microbial biomass in our tested systems than in degraded soils and the better aeration conditions than in anaerobic sediments could be the main reasons.

By contrast, 1678 and 1874 mg lignin kg^−1^ soil were accumulated in upland and upland-paddy, respectively, within 13 years’ straw incorporation (Fig. 2). The dead roots only contributed 114–136 mg kg^−1^ to lignin accumulation as calculated from the treatment without straw, indicating that the accumulated lignin mainly derived from the incorporated crop straw. The accumulation of lignin was significantly faster than that of total SOM (Table 1), highlighting the recalcitrant nature of lignin as one of the most abundant constituents in rice straw that has a potential to contribute to C sequestration.

Unlike the cellulose, lignin showed a higher annual accumulation rate in upland-paddy rotation but not in upland (Fig. 2). This is contrary to the hypothesis that higher lignin could accumulate in the upland which had higher SOC stock as compared with upland-paddy rotation. Although the dry-wet cycles could stimulate microbial activity and increase SOM mineralization^35,36^, the SOM decomposition was retarded under anaerobic conditions during rice season^3,37^. The upland-paddy rotation system was flooded for rice plantation in July after three months of straw incorporation. However, all of the newly added cellulose was converted into other non-cellulose forms in the upland-paddy before this time (Fig. 1a). Thus, cellulose would not accumulate during the flooded period, while the accumulation of recalcitrant components, e.g. lignin, might benefit from the anaerobic condition. Besides, the lower laccase enzyme activity in upland-paddy than in upland could also contribute to the higher accumulation rates of lignin (Fig. 4b; Fig. 5).

The dominant lignin monomers for both treatments were switched from V-type to C-type in upland or both V- and C-types in upland-paddy after 13 years’ cultivation, indicating a distinctly proportional increase of C monomer and a decrease of V monomer (Fig. 2). Because the V-type lignin was more stable than the S- and C-type lignin^38^, the stability of lignin was decreased after the land was reclaimed as arable land, especially in upland (Fig. 2). The dominant C-type lignin in both systems could be attributed to the fact that lignin was mainly derived from gymnosperm (V-type lignin) before 2000 (see Materials and methods), but its main source was changed to crop residues (C-type lignin)^38^ after land reclamation. The higher proportion of V-type lignin in upland-paddy than in upland, indicating the higher stability of lignin in the former system.

Similar to the dynamics of cellulose and lignin in degraded soils under a semiarid climate (deduced from Fig. 1 of the reference^16^), the cellulose and lignin contents were slightly increased in the last season for some treatments in this study (Figs 1 and 2). Some wild herbaceous plants naturally grown in spring in the PVC tubes. Although most of the plant biomass was removed by hand, the dead roots might contribute to the total lignin accumulation^39^. In this study, we only determined the total VSC-lignin in soils, the actual newly added lignin from crop straw, the lignin in intrinsic soil, and the lignin from dead root of wild plants in soil could not be differentiated.

The fate of C is, to a major extent, regulated by the availability of other key nutrient elements in arable soils^4^. In this study, lignin contents increased from 189–415 to 1972–2092 mg kg^−1^ with an increase in total N (from 0.67–0.76 to 0.91–1.02 g kg^−1^ DW soil; Table 1) after 13 years’ straw incorporation. SEM analysis identified the AN was the key driver of the dynamics of labile and recalcitrant organic materials, e.g. cellulose and lignin (Fig. 5). Thus, the increasing N availability was particularly important to increase SOM stock^16^. Much evidence showed that N could accelerate the transformation of the light-fraction of SOM, but suppress the transformation of the heavy fractions^40,41^. Here, we showed that the accumulations of both cellulose and lignin were driven by AN (Fig. 5). However, our data could not answer the questions how N affect cellulose and lignin decomposition and to what direction and extent it can influence, because we did not consider the treatments without N supplement in our study. Nitrogen exerts direct and indirect influence on lignin dynamic via multiple pathways (Fig. 5), and mainly via influencing the microbial processes^42–44^. (1) Microorganisms (bacteria and fungi) that biodegrade lignin are known to be suppressed by N content due to the reduced demand to mine the recalcitrant fractions^16,42^. (2) Nitrogen supplement was found to repress the synthesis of ligninolytic enzymes, e.g. phenol oxidase^45^, and to down-regulate expression of ligninolytic gene and reduce laccase activity^40,46^, although the activity of laccase (one of the phenol oxidase) had a positive relationship to AN (Fig. 5c; Fig. 5d). (3) Nitrogen supplement significantly accelerated the decomposition of light soil C fractions, but stabilized soil C compounds in heavier, mineral-associated fractions^47^. The dynamics of AN could be partly attributed to the soil pH (Fig. S3) and water regimes in the two systems^48,49^.

The effects of soil pH on cellulose and lignin dynamics were limited to an indirect effect of N availability in upland, or indirect influence of enzyme activity and microbial gene abundance in upland-paddy (Fig. 5 and S3). Many previous studies demonstrated that soil pH serves as the main driving factor for the microbial community and activity, especially bacteria^50^, and thus controlling C substrate utilization^51^.

The enzymes of cellobiohydrolases and laccases associate with the degradation of cellulose and lignin, respectively^52^. The SEM analysis showed the cellobiohydrolases activity was not the key driver of cellulose dynamic (Fig. 5a, Fig. 5b), while laccases exert direct effects on lignin dynamics in both tested systems (Fig. 5c, Fig. 5d). It indicated that cellobiohydrolases might not be the key enzymes involving in cellulose transformation, but laccase could be a good enzymatic predictor for lignin transformation in the tested soils. The abundance of the microbial *cbh*I gene had a significant correlation to cellulose dynamic in upland-paddy but not in upland, and a similar phenomenon for the *laccase*-like gene (Fig. S1), suggesting the predictor of the microbial functional gene in various soil ecosystems might be different^45,53,54^.

## Conclusion

The combination of *in situ* long- and short-term field experiments provided comparatively comprehensive data to recognize the dynamics of the main components (cellulose and lignin) of exogenous organic materials after straw was incorporated into arable soils. Our findings suggest straw incorporation favors the accumulation of recalcitrant components, e.g. lignin, but not relatively labile components, e.g. cellulose, in subtropical upland and upland-paddy soils. The loss of cellulose in upland-paddy is faster than in upland, but the opposite trend has been found for lignin, indicating a different response of relatively labile and recalcitrant components of plant residues to cropping systems. Although the patterns of cellulose and lignin degradation genes in upland and upland-paddy soils have not been consistent, higher cellobiohydrolases activity in upland-paddy relative to upland soils suggested a faster decomposition rate of cellulose in upland-paddy systems. Available nitrogen is the key direct driver for the dynamics of both cellulose and lignin in both soils based on SEM analysis.

## Methods

### Site description

Two adjacent (50 m away) long-term straw incorporation experiments involving two management practices (upland and upland-paddy rotation systems) were established in 2000 at a hilly site (29°13′48″ N, 111°31′36″ E) in Taoyuan County, Hunan Province of the subtropical China. This area is characterized by a subtropical humid monsoon climate, with a mean annual rainfall of 1,330 mm and mean annual air temperature of 16.8 °C. The soil is a clay loam (Ultisol, USDA soil taxonomy) developed from a Quaternary red earth. *Cunninghamia lanceolata* (Lamb.) Hook dominated in the experimental site until 1999.

Since 03/2000 (Month/Year), the experimental field was reclaimed for agricultural land uses. Simultaneously, a field experiment involved in two cropping systems was established, i.e. a rotation of sweet potato and rapeseed for upland, and maize, rice, and green manure (*Astragalus sinicus*) for the upland-paddy rotation^8^. Both cropping systems were plowed twice each year. The upland-paddy system was flooded on 19^th^ July for rice plantation and dried in 26^th^ October for rice harvest in 2013. There were four fertilization treatments for each cropping system as introduced in Zhu *et al*.^11^. In a random field arrangement, each treatment contained four replicate plots (3.9 m × 6.0 m). In this study, two fertilization treatments were selected for each cropping system. The treatment without straw was fertilized by chemical fertilizers (NPK; 224 kg N, 52 kg P, and 174 kg K ha^−1^ yr^−1^ as urea, superphosphate, and potassium chloride, respectively). The treatment with straw was fertilized by chemical fertilizers (137 kg N and 36 kg P ha^−1^ yr^−1^ as urea and superphosphate) and rice straw at the rate of 12,700 kg ha^−1^ yr^−1^ (contained 390 g C kg^−1^, 6.85 g N kg^−1^, 1.26 g P kg^−1^, and 13.7 g K kg^−1^)^11^. The annual N, P, and K supplements were thus equal between the treatments with straw and without straw. The straw was annually applied in two equal parts for sweet potato and rapeseed (upland) or maize and rice (upland-paddy).

### Experimental design and soil sampling

On 23/04/2013 (Date/Month/Year), five soil cores (3.5-cm diameter) within one plot were collected at 0–15 cm depth and homogenized as one composite sample. On 25/04/2013, five columns (35-cm height and 25-cm diameter polyvinylchloride tubes) were inserted into 28 cm soil depth of each replicate plot (no plant in the tubes). Surface soils (0–15 cm) from the columns were removed to plastic trays and then mixed with half amount of fertilizer and straw at their annual rates. The mixed soils were then put back to the corresponding column. During the experiment, the columns were covered by white nylon nets to prevent the entry of fresh crop residue. To decrease the influence of artificial-disturbance, the first-time soil sample was collected after three days. ~100 g soil from 0−15 cm depth of each column was collected using a stainless steel auger (Ǿ 3 cm) on 28/04/2013 and then every three-month on 28/07/2013 (the upland-paddy system was flooded), 28/10/2013, 28/01/2014 and 28/04/2014. Soil samples from the five columns within each plot were mixed as one composite sample. The applied straw was collected from an adjacent field site, oven dried (70 °C) and stored. Stem internode material was separated and used to ensure uniformity of the material, as internode, node and leaf material may vary in the content of cellulose and lignin. The straw was cut into pieces (approximately 2 mm), thoroughly mixed and sterilized by gamma irradiation. After soils collection, the holes excavated by stainless steel auger were gently filled by their surface soil from the columns to prevent waterlogging.

All soil samples were separated into three parts. The first part (~20 g) was packed into a sterile bag and immediately immersed in liquid N and transported to the laboratory. These samples were freeze-dried and stored in 10-mL sterile centrifuge tubes at −70 °C for further microbial gene abundance analysis. The second part (~50 g) was stored at 4 °C for enzyme activity analysis. The third part (~30 g) was air-dried and ground for analysis of cellulose, lignin, and other physicochemical properties.

### Basic soil property analyses

Soil pH was determined using 1:2.5 (w/v) soil-to-water ratio extracts. Determination of SOM, total N (TN), total phosphorus (TP), and total potassium (TK) was according to the dichromate oxidation^55^, the Kjeldahl method^56^, the NaOH fusion^57^, and the NaOH fusion-flame photometry method^58^, respectively. Available nitrogen (AN) was determined according to the alkali-hydrolytic diffusion method^59^. Available phosphorus (AP) was extracted with 0.5 M sodium bicarbonate (pH 8.5) and determined by the Mo–Sb colorimetric method^60^. Available potassium (AK) was extracted with 1.0 M ammonium acetate (pH 7.0) and determined with the flame photometry method^61^. The basic soil physicochemical properties before (in 2000) and after the long-term straw treatments (in 2013) are shown in Table 1. Soil microbial biomass C (MBC) was measured by the Fumigation-Extraction method^62^. Briefly, portions of wet soil (equivalent to about 20 g of oven-dried soil) were fumigated by exposing the soil to alcohol-free CHCl_3_ vapor for 24 h in a vacuum desiccator. The residual CHCl_3_ was removed by vacuuming 3−8 times, each for about 5 min. Then, the wet fumigated and non-fumigated soils were extracted with 80 ml of 0.5 M K_2_SO_4_ by shaking at 250 rpm for 30 min. The suspensions were filtered through Whatman No. 42 filter papers. Organic C in the extracts was analyzed by an automated procedure using a total C analyzer (Phoenix 8000, USA). The amount of MBC was calculated from the amounts of total organic C determined in the fumigated soil minus those in the non-fumigated soil, using a conversion factor (kc) of 0.45.

### Contents of cellulose and lignin

Cellulose in the soil was calculated by subtracting the dilute acid-hydrolyzed carbohydrates (0.5 M H_2_SO_4_) from the total carbohydrates hydrolyzed using the concentrated sulfuric acid (12 M H_2_SO_4_) according to their specific procedures^63,64^. Lignin in the soil was quantified by the alkaline CuO oxidation of the samples to release lignin monomers, followed by gas chromatography according to the method developed by Hedges *et al*.^65^, modified by Kögel *et al*.^66^ and described in detail by Liu *et al*.^67^. Lignin content was calculated as the sum of vanillyl (V), syringyl (S), and cinnamyl (C) type phenols^68^.

### Enzyme activity: cellobiohydrolases and laccases

Cellobiohydrolases (EC 3.2.1.91; alt. cellulose 1,4-beta-cellobiosidase) activity in soil was fluorimetrically measured by the methylumbelliferone-labeled substrate (4-MUB-d-cellobioside) using a microplate fluorometer (TECAN Infinite 200; Crailsheim, Germany) at 365 nm excitation and 450 nm emission wavelength, respectively^69^. Soil laccases activity (one of the key enzymes to decompose lignin) was spectrophotometrically measured (U-2000; Hitachi Ltd, Tokyo, Japan) using 2,2-azino-bis (3-ethylbenzothiazoline-6-sulfonate) (ABTS; Sigma) as the substrate^70^.

### Abundance of microbial genes

Microbial DNA was extracted from 0.5 g lyophilized soils with the FastDNA Spin Kit (BIO101; Vista, CA, USA) according to the manufacturer’s instructions. The quality and quantity of DNA were evaluated using a spectrophotometer ND-1000 (Nanodrop, PeqLab, Germany). The abundances of the *cbh*I gene (for cellulose decomposition) and *laccase*-like gene (for lignin decomposition) were determined using the real-time polymerase chain reaction (PCR; ABI 7900; Foster City, CA, USA). The information of primers, thermal conditions, and standard curves were shown in Table S2. The efficiency of all reactions ranged from 85% to 110%. The PCR was performed by placing standard curve samples and samples with or without template DNA on a 384-well plate. The copies of a target gene in the reaction mixtures of soils were automatically analyzed using the SDS 2.3 software. The abundance of target genes (copies g^−1^ DW soil) was calculated from the copies calibrated with a factor converting the DNA concentration from the soil DNA templates (5 ng μL^−1^ DNA) to per gram of DW soil (ng g^−1^ DW soil).

### Statistical analyses

Data (mean ± SE, n = 4) of cellulose, lignin and their proportions in SOM among treatments at each sampling time were subjected to standard one-way variance (ANOVA), and significant differences were compared by the least significant differences (LSD) test at *P* < 95% using the SPSS 18.0 software for Windows (SPSS Inc., Chicago, IL, USA). Homogeneity of variances was tested by Levene’s test, normal distribution of residues was tested by Shapiro-Wilk test. Statistical testing the effect of sampling time on parameters was performed using repeated measures ANOVA.

The structural equation modeling (SEM) framework was applied to investigate direct and indirect effects of enzyme activities, microbial gene abundances, pH and soil physicochemical properties on the amounts of cellulose or lignin. To simplify the model, only AN and pH was used to represent soil physicochemical properties because they had been shown as major predictors of variance in cellulose and lignin in an earlier running of both the Pearson correlation analysis and stepwise linear regression analysis. The SEM was carried out by the Amos 17.0 software package (Small waters Corporation, Chicago, IL, USA). Meanwhile, *P*-values and χ^2^ values were used to test the SEM fit, and the high *P-*values (*P* > 0.05) but small χ^2^ values indicated that the data fit well with the model. The goodness-of-fit index (GFI) and the root mean square error of approximation (RMSEA) were also reported, considering that the χ^2^ value was influenced by sample size. A GFI value that was higher than 0.9 and a RMSEA value lower than that was 0.07 suggested a significant fit to the model^71^.

## Electronic supplementary material

Supplementary Tables and Figures
