# Supplementary material for: Lignin and cellulose dynamics with straw incorporation in two contrasting cropping soils
Source: Sci Rep. 2018 Jan 26;8:1633. doi: 10.1038/s41598-018-20134-5 (PMC5786085; doi:10.1038/s41598-018-20134-5)
Supplement: Supplementary file 1 — Supplementary Tables and Figures [file 41598_2018_20134_MOESM1_ESM.pdf]

Lignin and cellulose dynamics with straw incorporation in two contrasting cropping soils

Xiangbi Chen[1,2], Yajun Hu[1,2], Shuzhen Feng[1,3], Yichao Rui[4], Zhenhua Zhang[2], Hongbo He[5], Xinhua He[6], Tida Ge[1], Jinshui Wu[1], Yirong Su[1 *]

[1] Key Laboratory of Agro-ecological Processes in Subtropical Region, Institute of Subtropical Agriculture, the Chinese Academy of Sciences, Changsha 410125, PR China. [2] Southern Regional Collaborative Innovation Center for Grain and Oil Crops in China, Hunan Agricultural University, Changsha 410128, PR China. [3] Guangxi University of Science and Technology, Liuzhou 545006, PR China. [4] Department of Soil Science, University of Wisconsin-Madison, Madison, WI 53706, U.S. [5] Key Laboratory of Terrestrial Ecological Process, Institute of Applied Ecology, the Chinese Academy of Sciences, Shenyang 110016, PR China. [6] Centre of Excellence for Soil Biology, College of Resources and Environment, Southwest University, Chongqing 400715, PR China. Correspondence and requests for materials should be addressed to Yirong Su (e-mail: yrsu@isa.ac.cn).

Table S1 Indices of fit for the structural equation models given in Fig 6. dF=degrees of freedom, χ^2=chi-square (minimum function test statistic), RMSEA=root mean square error of approximation.

No. Figure	Fit index									
	χ^2	*p*	dF	CFI	NFI	IFI	RMSEA	AIC	BCC	ECVI
Fig.6A	0.092	0.761	1	1.000	0.999	1.007	0.000	28.092	32.190	0.598
Fig.6B	0.682	0.409	1	1.000	0.995	1.002	0.000	26.682	32.779	0.610
Fig.6C	0.101	0.750	1	1.000	0.999	1.006	0.000	28.101	32.199	0.598
Fig.6D	1.478	0.224	1	0.995	0.987	0.996	0.101	29.478	33.575	0.627

Table S2 Primers and thermal profiles used for real-time PCR quantification of different microbial genes.

Target gene	Primers	Thermal profile	Range of standard curve
*cbh*I	fungcbhIF/fungcbhIR	1 cycle of 94°C for 2min；40 cycles of 94°C for 30s，48°C for 45, 72°C for 60s; 1 cycle of dissociation (95°C for 15s, 60°C for 15s, 95°C for 15s)	$3.01 \times 10^2 – 3.01 \times 10^9$
laccase-like	Cu1AF/Cu2R	1 cycle of 95°C for 30s；40 cycles of 95°C for 5s，55°C for 30s; 1 cycle of dissociation (95°C for 15s, 65°C for 15s, 95°C for 15s)	$3.92 \times 10^2 – 3.92 \times 10^9$

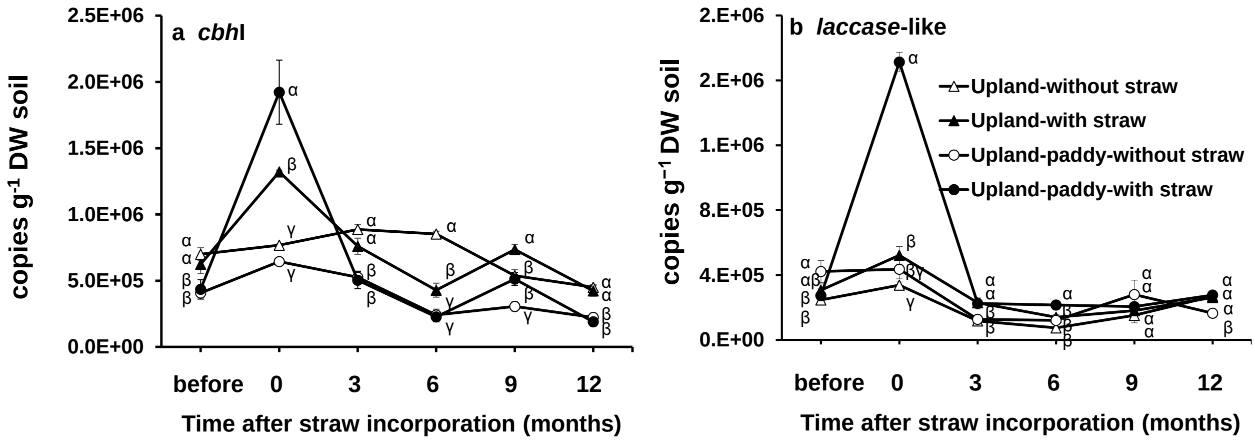

Fig. S1. Dynamics of the abundances of *cbh*I (A), *laccase*-like genes (B) (means ± SE, n = 4). Before means the day before straw incorporation in 2013. Different *Greek letters* indicate significant difference among treatments at each sampling time at $P < 0.05$ level (according to one-way-ANOVA and LSD test).

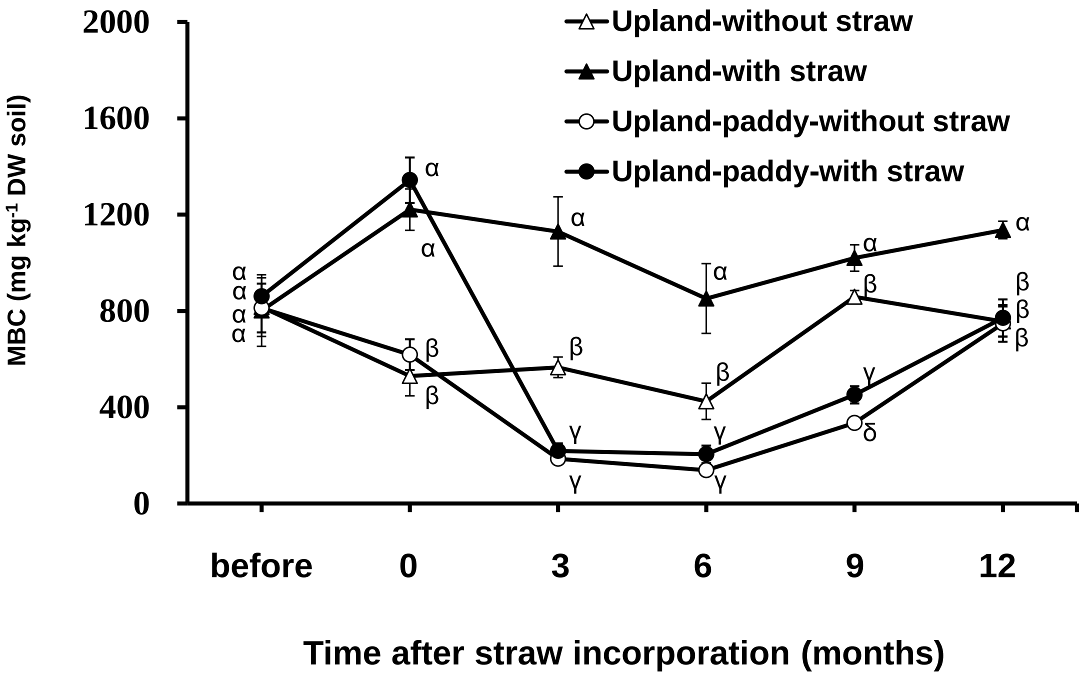

Fig. S2. Dynamics of microbial biomass carbon (MBC) over a one-year period of straw incorporation (means ±SE, n=4). before means the day before straw incorporated in 2013. Different *Greek letters* indicate significant difference among treatments at each sampling time at $P < 0.05$ level (according to one-way-ANOVA and LSD test).

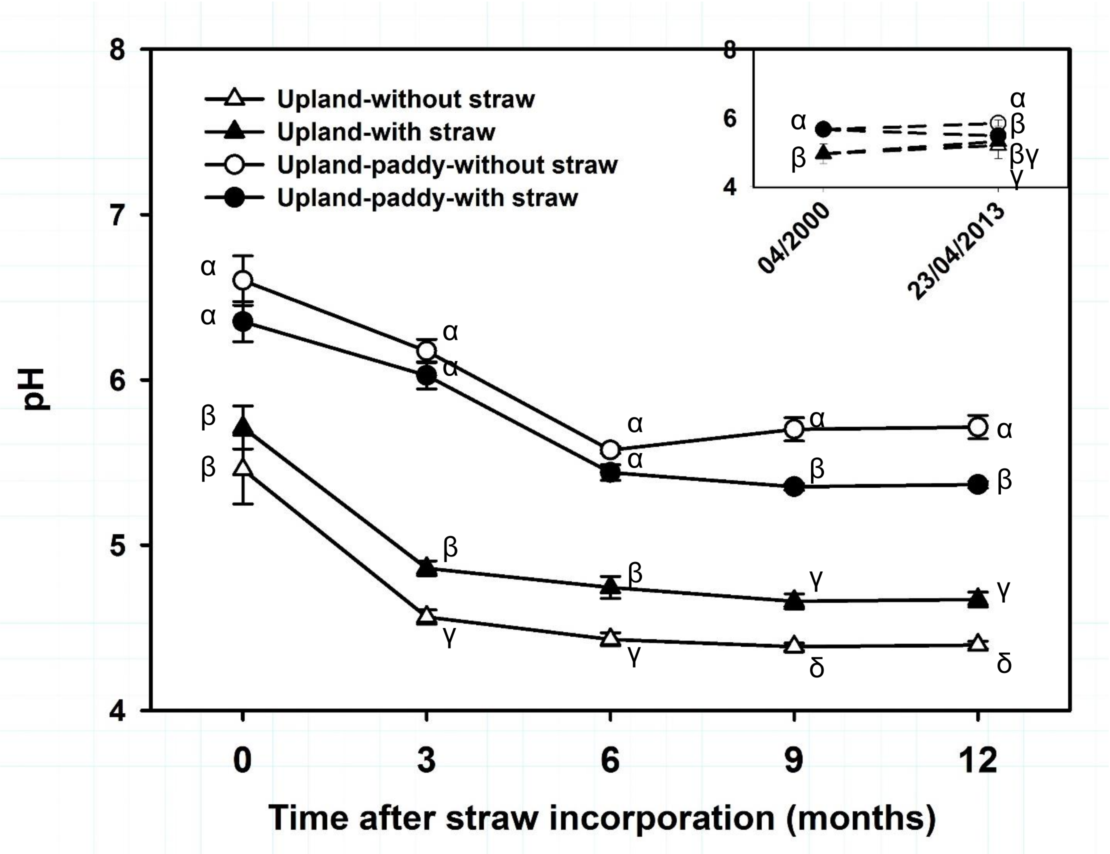

Fig. S3 Dynamics of soil pH over a one-year period of straw incorporation in the tested upland and upland-paddy soils (means ±SE, n=4). 04/2000, the time before the field experiment was set; 23/04/2013, the day before straw incorporated in 2013. Different *Greek letters* indicate significant difference among treatments at each sampling time at $P < 0.05$ level (according to one-way-ANOVA and LSD test).
